# Expression of miRNA-106b in conventional renal cell carcinoma is a potential marker for prediction of early metastasis after nephrectomy

**DOI:** 10.1186/1756-9966-29-90

**Published:** 2010-07-07

**Authors:** Ondrej Slaby, Jana Jancovicova, Radek Lakomy, Marek Svoboda, Alexandr Poprach, Pavel Fabian, Leos Kren, Jaroslav Michalek, Rostislav Vyzula

**Affiliations:** 1 Masaryk Memorial Cancer Institute, Department of Comprehensive Cancer Care, Zluty kopec 7, Brno, Czech Republic; 2 Masaryk Memorial Cancer Institute, Department of Oncological and Experimental Pathology, Zluty kopec 7, Brno, Czech Republic; 3 Babak Research Institute, University Cell Immunotherapy Center, Kamenice 5, Brno, Czech Republic; 4 Masaryk University, Faculty of Science, Department of Biochemistry, Kotlarska 2, Brno, Czech Republic; 5 University Hospital Brno, Department of Pathology, Faculty of Medicine, Masaryk University, Brno, Czech Republic

## Abstract

**Background:**

MicroRNAs are endogenously expressed regulatory noncoding RNAs. Previous studies have shown altered expression levels of several microRNAs in renal cell carcinoma.

**Methods:**

We examined the expression levels of selected microRNAs in 38 samples of conventional renal cell carcinoma (RCC) and 10 samples of non-tumoral renal parenchyma using TaqMan real-time PCR method.

**Results:**

The expression levels of miRNA-155 (p < 0.0001), miRNA-210 (p < 0.0001), miRNA-106a (p < 0.0001) and miRNA-106b (p < 0.0001) were significantly over-expressed in tumor tissue, whereas the expression of miRNA-141 (p < 0.0001) and miRNA-200c (p < 0.0001) were significantly decreased in RCC samples. There were no significant differences between expression levels of miRNA-182 and miRNA-200b in tumor samples and renal parenchyma. Our data suggest that expression levels of miRNA-106b are significantly lower in tumors of patients who developed metastasis (p = 0.030) and miR-106b is a potential predictive marker of early metastasis after nephrectomy in RCC patients (long-rank p = 0.032).

**Conclusions:**

We have confirmed previous observations obtained by miRNA microarray analysis using standardized real-time PCR method. For the first time, we have identified a prognostic significance of miRNA-106b, which, after validation on a larger group of patients, maybe useful as a promising biomarker in patients with RCC.

## Background

Renal cell carcinoma (RCC) accounts for approximately 3% of cancers in adults as well as 85% of all primary malignant kidney tumors. It is the third most common urological cancer after prostate and bladder cancer but it has the highest mortality rate at over 40% [1,2]. Clear cell (conventional) carcinoma is the most common subtype of RCC and accounts for approximately 75-80% of these tumors [3]. Apart from surgery, it is both chemotherapy and radiotherapy resistant. The present absence of biomarkers for early detection and follow-up of the disease is responsible for late diagnosis and subsequent poor prognosis. It is necessary, therefore, to improve our understanding of RCC's pathogenesis, identify new biomarkers enabling prediction of early metastasis after nephrectomy, and develop new targeted therapies.

One of the most modern and progressive approaches for molecular characterization of tumors today is based on microRNA expression profiles. MicroRNAs (miRNAs) are short noncoding RNAs, 18-25 nucleotides in length, that post-transcriptionally regulate gene expression. Depending upon the extent of their complementarity with target mRNA, miRNAs act by two mechanisms of post-transcriptional regulation of gene expression, which lead to target mRNA degradation or repression of its translation and consequent decrease of particular protein levels. Bioinformatics have predicted that miRNAs have the capacity to regulate one third of all mammalian genes, among which are included a significant number of important oncogenes and tumor suppressor genes [4,5]. MiRNAs have been studied most intensively in the field of oncological research, and emerging evidence suggests that altered miRNA regulation is involved in the pathogenesis of cancer [6-8]. Changes in the expression of miRNAs have been observed in a variety of human cancers [9-11].

Several studies have focused on miRNAs' significance in RCC [12]. These papers described the potential of miRNA profiles to distinguish tumor tissue from normal renal parenchyma [13-20], classify renal cell carcinomas according to histological subtypes [13-15], identify expression profiles to predict metastasis from primary tumors [13,16], and determine prognosis for particular renal cell carcinoma patients [13,16]. Most of these studies have been methodologically based on hybridization microarray platforms and mostly included only small numbers of patients. Nevertheless, there exists a certain intersection between groups of miRNAs identified in individual studies, and several interesting mechanistic studies have revealed the functions of some miRNAs in vitro [21].

The aim of our study was to validate expression changes of selected miRNAs identified in previous microarray studies (miR-155 [16], miR-106a [19], miR-106b [19], miR-200b [16,19], miR-200c [15,16,19], miR-141 [15,16], miR-182 [13] and miR-210 [16,19]) by the standardized and more quantitative method that is real-time polymerase chain reaction (PCR). For the first time, we have correlated miRNAs with the relapse-free survival of RCC patients in order to evaluate them as potential predictive biomarkers of early metastasis after nephrectomy.

## Patients and methods

### Study population

Thirty-eight patients (24 men, 14 women) diagnosed for clear cell renal cell carcinoma at Masaryk Memorial Cancer Institute (Brno, Czech Republic) between 2003 and 2009 were included in this study. The study has been approved by the local Ethical Committee. Patients' ages ranged between 41 and 89 years, with a median of 68. Histological diagnosis was established according to the guidelines of the World Health Organization. Cases were selected according to tissue availability and were not stratified for any known preoperative or pathological prognostic factor. Clinical follow-up data in the form of annually assessed survival time was available for all patients. The median follow-up time for all cases was 40 months and ranged from 3 to 105 months. Clinical characteristics of the patients are summarized in Table 1.

**Table 1:** Patient characteristics

Factor	Number
**Age**	
mean	68
range	41-89
**Sex**	
male	24
female	14
**Stage**	
T1+T2	19
T3	19
**Fuhrman grade**	
G1	6
G2	25
G3	7
**Early recurrence**	
Yes*	15
No**	23

* Recurrence-free survival = 11.5 (5-36) months

** Follow-up = 50 (41-62) months

Medians and 25th and 75th percentiles in parentheses.

### Tissue sample preparation and miRNA purification

Under the supervision of an experienced pathologist, 48 tissue samples were collected (before any treatment was started) from surgically resected tissues - 38 samples from primary tumors and 10 from adjacent non-tumoral renal parenchyma. All samples were immediately stored in liquid nitrogen until RNA extraction. Samples were homogenized (Retch MM301) in sterile conditions before total RNA isolation. Total RNA isolation and small RNA enrichment procedures were performed using the mirVana miRNA Isolation Kit (Ambion, USA) according to the manufacturer's instructions. DNA was extracted using the Qiagen DNA Mini Kit (Qiagen, Germany), again following the manufacturer's instructions. Nucleic acid concentration and purity were controlled by UV spectrophotometry (A260:A280 > 2.0; A260:A230 > 1.8) using a Nanodrop ND-1000 (Thermo Scientific, USA).

### Real-time quantification of miRNAs by stem-loop RT-PCR

Complementary DNA (cDNA) was synthesized from total RNA using gene-specific primers according to the TaqMan MicroRNA Assay protocol (PE Applied Biosystems, Foster City, Calif., USA). Reverse transcriptase (RT) reactions utilized 10 ng of RNA sample, 50 nM of stem-loop RT primer, 1 × RT buffer and 0.25 mM each of dNTPs, 3.33 U/μl MultiScribe RT and 0.25 U/μl RNase inhibitor (all from the TaqMan MicroRNA Reverse Transcription kit of Applied Biosystems; 4366597). Reaction mixtures (15 μl) were incubated in a TGradient thermal cycler (Biometra) for 30 min at 16°C, 30 min at 42°C, 5 min at 85°C, and then held at 4°C. Real-time PCR was performed using the Applied Biosystems 7500 Sequence Detection System. The 20-μl PCR reaction mixture included 1.3 μl of RT product, 1 × TaqMan (NoUmpErase UNG) Universal PCR Master Mix, and 1 μl of primer and probe mix of the TaqMan MicroRNA Assay protocol (PE Applied Biosystems). Reactions were incubated in a 96-well optical plate at 95°C for 10 min, followed by 40 cycles at 95°C for 15 s and 60°C for 10 min. The threshold cycle data were determined using the default threshold settings. All real-time PCR reactions were run in triplicate and average threshold cycle (C_T_) and SD values were calculated.

### Data normalization and statistical analysis

Expression data were normalized according to expression of the RNU6B reference DNA (Assay No. 4373381; Applied Biosystems). Statistical differences between miRNA levels in RCCs and RP and differences in therapy response in relation to miRNA levels were evaluated using the nonparametric Mann-Whitney U test between 2 groups. Survival analyses were performed using the long-rank test and Kaplan-Meier plots approach. All calculations were performed using Statistica software version 6.0 (StatSoft Inc., USA).

## Results

We identified gene expression levels of the studied miRNAs in 38 RCCs and 10 non-tumoral renal parenchyma (RP). Differences between the two groups were evaluated using the Mann-Whitney test and also by the Wilcoxon test for ten paired samples. Both methods identified highly significant differences between RCC and RP in the expression levels of the most studied miRNAs. Significance levels and medians of the relative expression values with their ranges defined by the 25th and 75th percentiles are presented in Table 2. The real-time PCR analysis indicated no significant difference between RCC and the RP in expression levels of miR-200b and miR-182. By contrast, the expression levels of miR-155, miR-210, miR-106a and miR-106b were significantly upregulated in the tumor compared to the RP. The most significant difference was seen for miR-210, for which the expression levels were more than 60 times higher in RCC tissue. Conversely, miR-141 and miR-200 were significantly downregulated in RCCs (Table 2). The most significant difference was observed in miR-141, with levels in RCCs approximately 15 times lower than in the RP.

**Table 2:** Expression levels of selected miRNAs in renal cell carcinoma (RCC) in comparison to renal parenchyma (RP)

	non-paired samples			paired samples		
	**RCC**	**RP**	**p-value**	**RCC**	**RP**	**p-value**
	**n = 38**	**n = 10**		**n = 10**	**n = 10**	

**miRNA-155**	12.261*	0.430	**< 0.0001**	17.882	0.430	**0.002**
	3.945-24.127**	0.180-0.811		9.234-30.451	0.179-0.811	
**miRNA-141**	0.107	1.937	**0.002**	0.296	1.937	**0.004**
	0.047-0.630	1.128-2.805		0.107-1.235	1.128-2.805	
**miRNA-210**	38.947	0.592	**< 0.0001**	28.264	0.592	**0.002**
	25.153-74.817	0.488-0.701		7.750-73.600	0.488-0.701	
**miRNA-200c**	0.268	2.41	**< 0.0001**	0.259	2.41	**0.002**
	0.171-0.508	1.910-3.408		0.171-0.565	1.910-3.408	
**miRNA-106a**	20.557	4.934	**< 0.0001**	22.846	4934	**0.004**
	13.302-31.403	3.857-7.910		13.302-28.127	3.857-7.910	
**miRNA-106b**	14.102	2.937	**< 0.0001**	16.356	2973	**0.002**
	8.807-20.746	1.941-3.963		12.101-30.239	1.942-3.963	
miRNA-200b	7.384	5.702	0.559	5.756	5702	0.846
	3.599-15.578	4.715-8.260		1.892-9.571	4.715-8.260	
miRNA-182	0.147	0.121	0.919	0.19	0.121	0.322
	0.047-0.515	0.086-0.154		0.038-0.491	0.086-0.154	

* Medians of expression level related to RNU6B with **25th and 75th percentiles.

To evaluate the potential association of individual miRNAs with the prognosis of RCC patients after nephrectomy, the expression levels of each miRNA in the group of patients who developed metastasis were compared with their levels in the group of patients in remission. Metastatic patients tended to have lower levels of miR-155, miR-106a and miR-106b in RCCs, but only miR-106b reached statistical significance (p = 0.03) (Table 3). To determine differences for relapse-free survival on the basis of miR-106b expression, Kaplan-Meier plots were constructed and the long-rank test was performed. This analysis demonstrated significant difference (p = 0.032). On the other hand, no association was found between the development of metastasis and miR-155, miR-210, miR-106a, miR-200b and miR-200c (Table 3).

**Table 3:** Expression levels in RCC of patients with/without development of metastatic disease

	Metastatic disease	Relapse-free disease	p-value
	n = 15	n = 23	
miRNA-155	6.07*	15.58	0.095
	1.80-21.03**	7.29-27.58	
miRNA-141	0.072	0.102	0.682
	0.033-0.671	0.046-0.181	
miRNA-210	36.758	41.137	0.317
	12.147-62.417	28.581-82.332	
miRNA-200c	0.266	0.301	0.502
	0.108-0.487	0.185-0.517	
miRNA-106a	14.522	25.126	0.081
	12.469-24.862	16.923-42.519	
**miRNA-106b**	10.387	16.451	**0.030**
	6.591-15.950	11.119-25.831	
miRNA-200b	6.556	7.804	0.87
	3.849-14.096	3.697-14.676	
miRNA-182	0.302	0.081	0.194
	0.051-0.728	0.036-0.321	

* Medians of expression level related to RNU6B with **25th and 75th percentiles.

## Discussion

It has become evident that genomic information for transcribing miRNAs is implemented in the human genome, and miRNAs constitute a robust regulatory network with post-transcription regulatory efficiency for almost one third of human coding genes. A number of different approaches to quantifying miRNAs in tumors and non-tumoral tissue have been described, including microarrays, modified invader assay, bead-based flow cytometric assay, and real-time PCR [11]. The main advantage of real-time PCR is the fact that it is a more quantitative and more sensitive method compared with other high-throughput assays. In our study, we have analyzed expression levels of selected miRNAs previously identified by global miRNA profiling studies in RCC clinical specimens as suspected diagnostic biomarkers using a standardized TaqMan real-time PCR approach on a larger group of RCC patients. This validation is necessary if one is to draw conclusions from the findings derived from hybridization microarray analysis.

One of the most frequently studied miRNAs in cancer biology, miR-155, has repeatedly been identified through miRNA microarray profiling as upregulated also in RCC tissue [15,16]. We have confirmed observations from these studies, inasmuch as miR-155 levels were almost 30 times higher in RCCs compared to RP. The available experimental evidence indicates that miR-155 is over-expressed in a variety of malignant tumors (breast, lung, colon, head/neck), which allows us to include this miRNA into the list of oncogenic miRNAs with high importance in cancer diagnosis and prognosis [22].

Three miRNA microarray studies have revealed downregulation of miR-141 and miR-200c in RCC tissue [15,16,18]. In agreement with these results, we have observed 20 times higher levels of miR-141 and 10 times higher levels of miR-200c in RP compared to RCCs. Both miR-200c and miR-141 are members of the miR-200 family that is mechanistically associated with the process of epithelial-mesenchymal transition (EMT). EMT is characterized by a decrease of E-cadherin, loss of cell adhesion, and increased cell motility leading to promotion of metastatic behavior of cancer cells (including RCC) [23]. A molecular link between EMT and the miR-200 family is represented by zinc-finger E-box binding homeobox 1 (ZEB1), a crucial inducer of EMT in various human tumors directly suppressing transcription of miR-141 and miR-200c, which strongly activate epithelial differentiation in pancreatic, colorectal and breast cancer cells [24]. On the other hand, ZFHX1B, also known as ZEB2 and Smad-interacting protein 1 (SIP1), was identified as the common target of miR-141 and miR-200c. It already has been reported that ZFHX1B is upregulated in a variety of human carcinomas and that it may function as a transcriptional repressor for E-cadherin [23].

Huang et al. [20] have described induction of miR-210 expression under the hypoxic conditions dependent on HIF-α expression. Mutations in the *VHL *gene, one of the key events in RCC pathogenesis, is associated with accumulation of HIF-α. Consistent with these findings and with previous profiling studies [16,19,20], we have observed more than 60 times higher levels of miR-210 in tumors.

We have identified significant upregulation of miR-106a and miR-106b in RCCs with approximately 4 and 5 times higher levels, respectively. Our data are in agreement with the results of Chow et al. [21], who used an approach based on real-time PCR. Interestingly, miR-106a and mirR-106b upregulation has not been detected by any other group focused on miRNA profiling in RCC [13-18], probably due to the lower sensitivity and lower dynamic range of hybridization-based microarrays. Over-expression of miR-106b, however, has been observed in a variety of human tumors, including colorectal cancer [25], gastric cancer [26], hepatocellular carcinoma [27] and head and neck squamous cell carcinomas [28].

We have not confirmed significant differences in miR-182 and miR-200b levels between RCCs and RP as reported by Petillo et al. [13], Jung et al. [16] and Chow et al. [19].

To date, only one study was done focusing on miRNAs' significance in RCC prognosis, and that involved a group of 8 RCC patients (4 patients indicated good and 4 poor prognosis) [13]. Petillo et al. [13] identified a group of 20 miRNAs enabling classification of RCC patients according to their prognosis. We have tested only one (miR-182) of these 20 miRNAs and have not proven its prognostic significance. Moreover, other analyzed miRNAs were evaluated as possible prognostic factors enabling the prediction of early metastasis after nephrectomy, and, except for miR-106b, none of these indicated significant potential to predict prognosis. Surprisingly, miR-106b, considered to be oncogenic [29], has significantly higher expression levels in RCC of patients with better prognosis. A possible explanation for this contradiction lies in the involvement of the miR-106b family (miR-106b, miR-93, and miR-25) in TGF-β signaling [30]. The role of TGF-β signaling in cancer pathogenesis is characteristically ambiguous [31]. In the early events of carcinogenesis, TGF-β levels are lower and indicate features of a tumor suppressor, but in the late phase, within the development of metastatic disease, the degree of TGF-β activation increases and leads to the promotion of immunosuppression, neoangiogenesis and progression of the disease. In relation to the TNM stage of RCCs, we have observed a general tendency for miR-106b levels to decrease from earlier stages towards advanced. Higher levels of miR-106b in selected RCCs may be connected with anti-neoplastic effects due to interference with TGF-β signaling.

## Conclusions

To our knowledge, this is the first report observing that the expression of miR-106b has a correlation with the development of metastasis and relapse-free survival in RCC patients after nephrectomy. Our findings also support the importance of miR-155, mR-210, miR-106a, miR-106b, miR-200c and miR-141 in RCC pathogenesis. While further studies and validations are needed, we suggest that miRNA-106b might be used for predicting early metastasis after nephrectomy in clinical practice. If validated, this would represent a next step to better treatment decisions and, ultimately, improvement in the survival rate of RCC patients.

## Abbreviations

RCC: renal cell carcinoma; miRNAs: microRNAs; RP: non-tumoral renal parenchyma; EMT: epithelial-mesenchymal transition; ZEB1: zinc-finger E-box binding homeobox 1; ZFHBX1: zinc finger homeobox 1B gene; SIP1: Smad-interacting protein 1; VHL: von Hippel-Lindau gene; HIF-α: hypoxia-induced factor alpha; TGF-β: transforming growth factor beta.

## Competing interests

The authors declare that they have no competing interests.

## Authors' contributions

JJ carried out most of the experiments and organized data for the manuscript. PF and LK performed histopathological diagnosis of clear cell renal cell carcinoma and participated in manuscript drafting. MS, RL, AP, JM and RV participated in data organization and manuscript drafting. OS performed project design, coordinated the study and writing of the manuscript. All authors read and approved the final manuscript.

**Figure 1:**
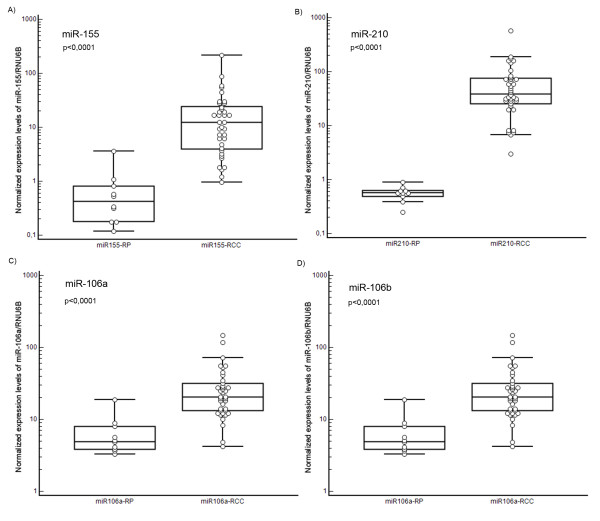
**Comparison of miR-155, miR-210, miR-106a and miR-106b expression levels in renal parenchyma (RP) and renal cell carcinomas (RCC)**.

**Figure 2:**
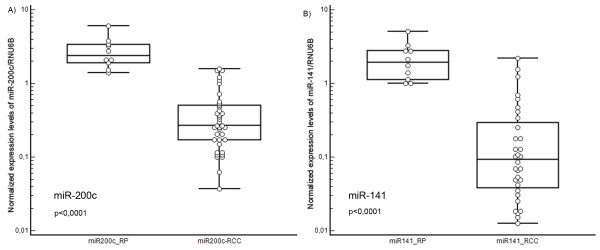
**Comparison of miR-200c and miR-141 (tumor suppressive miR-200 family) expression levels in RP and RCC**.

**Figure 3:**
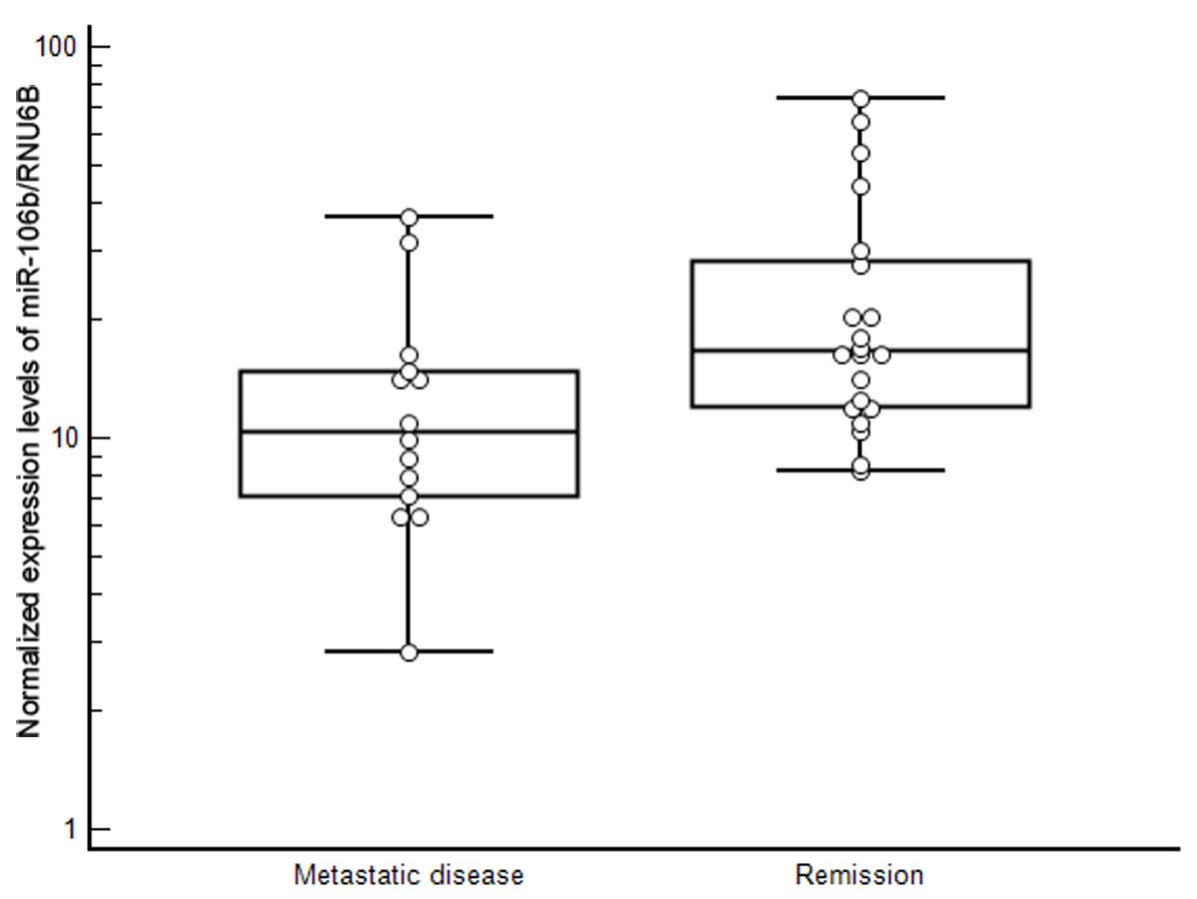
**Comparison of miR-106b expression levels in RCC stratified according to the development of metastatic disease after nephrectomy**.

**Figure 4:**
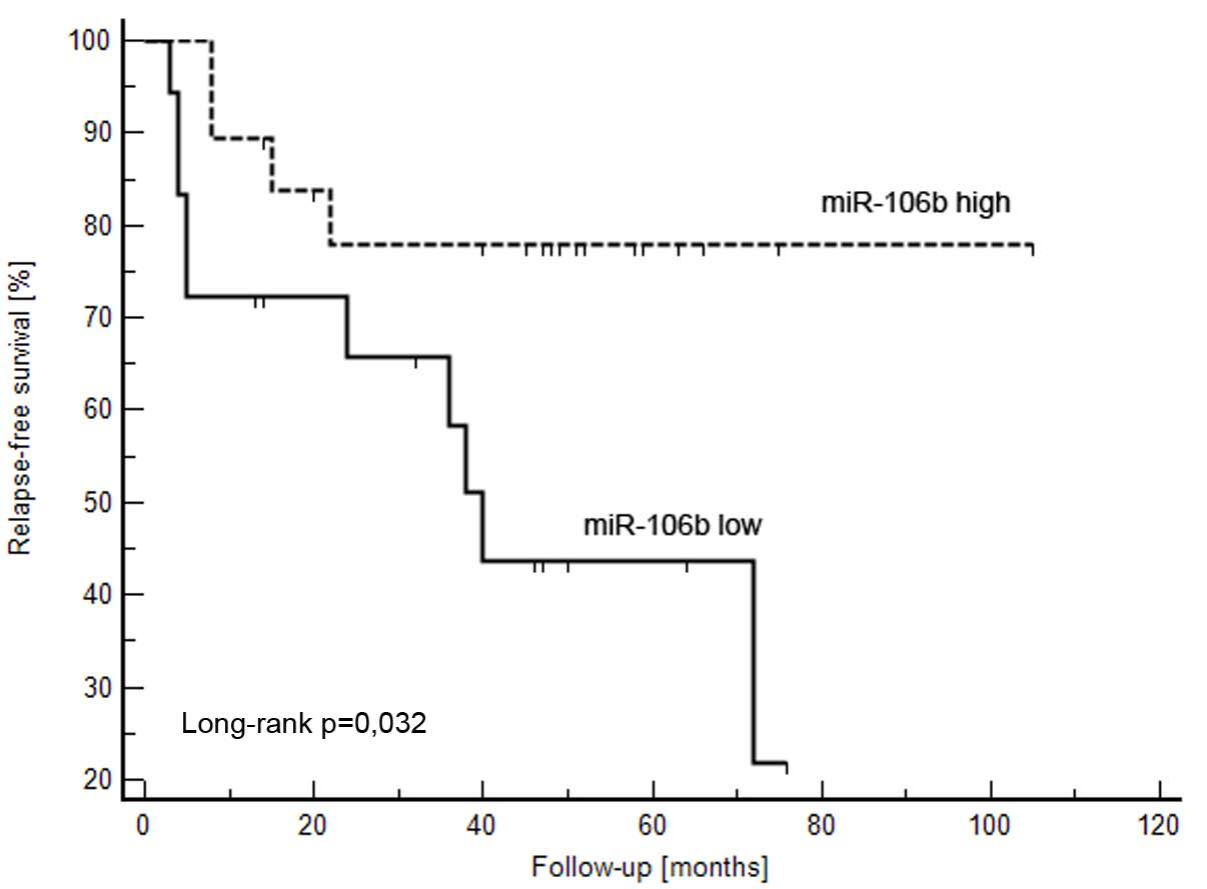
**Relapse-free survival of patients with RCC based on the miR-106b expression levels (cutoff = median of miR-106b expression)**.

## References

[B1] Richie JP Jonasch E Kantoff PW Kufe WD, Bast RC, Hait WN, et al Renal Cell Carcinoma Holland-Frei Cancer Medicine 2006 7 Hamilton (Canada), BC Decker 1401 1410

[B2] Bukowski RM Prognostic factors for survival in metastatic renal cell carcinoma: update 2008 Cancer 2009 115 2273 2281 10.1002/cncr.24226 19402065

[B3] Yan BC Mackinnon AC Al-Ahmadie HA Recent developments in the pathology of renal tumors: morphology and molecular characteristics of select entities Arch Pathol Lab Med 2009 133 102610 32 10.5858/133.7.1026 19642729

[B4] Inui M Martello G Piccolo S MicroRNA control of signal transduction Nat Rev Mol Cell Biol 2010 11 4 252 263 20216554 10.1038/nrm2868

[B5] Galasso M Elena Sana M Volinia S Non-coding RNAs: a key to future personalized molecular therapy? Genome Med 2010 18 2(2) 12 10.1186/gm133 PMC2847703 20236487

[B6] Brown BD Naldini L Exploiting and antagonizing microRNA regulation for therapeutic and experimental applications Nat Rev Genet 2009 10 578 585 10.1038/nrg2628 19609263

[B7] Bartels CL Tsongalis GJ MicroRNAs: novel biomarkers for human cancer Clin Chem 2009 55 623 631 10.1373/clinchem.2008.112805 19246618

[B8] Esquela-Kerscher A Slack FJ Oncomirs - microRNAs with a role in cancer Nat Rev Cancer 2006 6 259 269 10.1038/nrc1840 16557279

[B9] Garzon R Calin GA Croce CM MicroRNAs in Cancer Annu Rev Med 2009 60 167 179 10.1146/annurev.med.59.053006.104707 19630570

[B10] Garzon R Fabbri M Cimmino A Calin GA Croce CM MicroRNA expression and function in cancer Trends Mol Med 2006 12 580 587 10.1016/j.molmed.2006.10.006 17071139

[B11] Slaby O Svoboda M Michalek J Vyzula R MicroRNAs in colorectal cancer: translation of molecular biology into clinical application Mol Cancer 2009 8 102 10.1186/1476-4598-8-102 19912656 PMC2780389

[B12] Slaby O Svoboda M Michalek J Vyzula R DNA and microRNA microarray technologies in diagnostics and prediction for patients with renal cell carcinoma Klin Onkol 2009 22 5 202 209 19886357

[B13] Petillo D Kort EJ Anema J Furge KA Yang XJ Teh BT MicroRNA profiling of human kidney cancer subtypes Int J Oncol 2009 35 1 109 114 10.3892/ijo_00000318 19513557

[B14] Juan D Alexe G Antes T Liu H Madabhushi A Delisi C Ganesan S Bhanot G Liou LS Identification of a microRNA panel for clear-cell kidney cancer Urology 2010 75 4 835 841 10.1016/j.urology.2009.10.033 20035975

[B15] Nakada C Matsuura K Tsukamoto Y Tanigawa M Yoshimoto T Narimatsu T Nguyen LT Hijiya N Uchida T Sato F Mimata H Seto M Moriyama M Genome-wide microRNA expression profiling in renal cell carcinoma: significant down-regulation of miR-141 and miR-200c J Pathol 2008 216 4 418 427 10.1002/path.2437 18925646

[B16] Jung M Mollenkopf HJ Grimm C Wagner I Albrecht M Waller T Pilarsky C Johannsen M Stephan C Lehrach H Nietfeld W Rudel T Jung K Kristiansen G MicroRNA profiling of clear cell renal cell cancer identifies a robust signature to define renal malignancy J Cell Mol Med 2009 13 9B 3918 3928 10.1111/j.1582-4934.2009.00705.x 19228262 PMC4516539

[B17] Gottardo F Liu CG Ferracin M Calin GA Fassan M Bassi P Sevignani C Byrne D Negrini M Pagano F Gomella LG Croce CM Baffa R Micro-RNA profiling in kidney and bladder cancers Urol Oncol 2007 25 5 387 392 17826655 10.1016/j.urolonc.2007.01.019

[B18] Yi Z Fu Y Zhao S Zhang X Ma C Differential expression of miRNA patterns in renal cell carcinoma and nontumorous tissues J Cancer Res Clin Oncol 2010 136 6 855 862 10.1007/s00432-009-0726-x 19921256 PMC11827771

[B19] Chow TF Youssef YM Lianidou E Romaschin AD Honey RJ Stewart R Pace KT Yousef GM Differential expression profiling of microRNAs and their potential involvement in renal cell carcinoma pathogenesis Clin Biochem 2010 43 1-2 150 158 10.1016/j.clinbiochem.2009.07.020 19646430

[B20] Huang Y Dai Y Yang J Chen T Yin Y Tang M Hu C Zhang L Microarray analysis of microRNA expression in renal clear cell carcinoma Eur J Surg Oncol 2009 35 10 1119 1123 19443172 10.1016/j.ejso.2009.04.010

[B21] Chow TF Mankaruos M Scorilas A Youssef Y Girgis A Mossad S Metias S Rofael Y Honey RJ Stewart R Pace KT Yousef GM The miR-17-92 cluster is over expressed in and has an oncogenic effect on renal cell carcinoma J Urol 2010 183 2 743 751 10.1016/j.juro.2009.09.086 20022054

[B22] Faraoni I Antonetti FR Cardone J Bonmassar E miR-155 gene: a typical multifunctional microRNA Biochim Biophys Acta 2009 1792 6 497 505 19268705 10.1016/j.bbadis.2009.02.013

[B23] Gibbons DL Lin W Creighton CJ Rizvi ZH Gregory PA Goodall GJ Thilaganathan N Du L Zhang Y Pertsemlidis A Kurie JM Contextual extracellular cues promote tumor cell EMT and metastasis by regulating miR-200 family expression Genes Dev 2009 23 18 2140 2151 10.1101/gad.1820209 19759262 PMC2751985

[B24] Burk U Schubert J Wellner U Schmalhofer O Vincan E Spaderna S Brabletz T A reciprocal repression between ZEB1 and members of the miR-200 family promotes EMT and invasion in cancer cells EMBO Rep 2008 9 582 589 10.1038/embor.2008.74 18483486 PMC2396950

[B25] Wang YX Zhang XY Zhang BF Yang CQ Chen XM Gao HJ Initial study of microRNA expression profiles of colonic cancer without lymph node metastasis J Dig Dis 2010 11 1 50 54 10.1111/j.1751-2980.2009.00413.x 20132431

[B26] Guo J Miao Y Xiao B Huan R Jiang Z Meng D Wang Y Differential expression of microRNA species in human gastric cancer versus non-tumorous tissues J Gastroenterol Hepatol 2009 24 4 652 657 10.1111/j.1440-1746.2008.05666.x 19175831

[B27] Li Y Tan W Neo TW Aung MO Wasser S Lim SG Tan TM Role of the miR-106b-25 microRNA cluster in hepatocellular carcinoma Cancer Sci 2009 100 7 1234 1242 10.1111/j.1349-7006.2009.01164.x 19486339

[B28] Hui AB Lenarduzzi M Krushel T Waldron L Pintilie M Shi W Perez-Ordonez B Jurisica I O'Sullivan B Waldron J Gullane P Cummings B Liu FF Comprehensive MicroRNA profiling for head and neck squamous cell carcinomas Clin Cancer Res 2010 16 4 1129 1139 10.1158/1078-0432.CCR-09-2166 20145181

[B29] Petrocca F Vecchione A Croce CM Emerging role of miR-106b-25/miR-17-92 clusters in the control of transforming growth factor beta signaling Cancer Res 2008 68 20 8191 8194 10.1158/0008-5472.CAN-08-1768 18922889

[B30] Bierie B Moses HL Tumour microenvironment: TGFbeta: the molecular Jekyll and Hyde of cancer Nat Rev Cancer 2006 6 7 506 520 10.1038/nrc1926 16794634

[B31] Joshi A Cao D TGF-beta signaling, tumor microenvironment and tumor progression: the butterfly effect Front Biosci 2010 15 180 194 10.2741/3614 20036814

