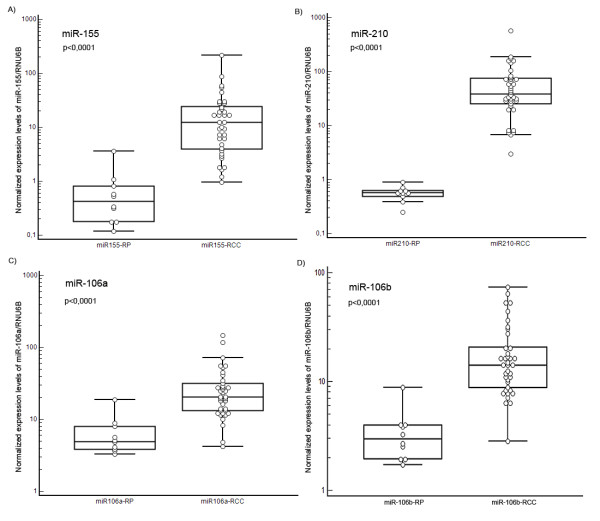# Expression of miRNA-106b in conventional renal cell carcinoma is a potential marker for prediction of early metastasis after nephrectomy

**DOI:** 10.1186/1756-9966-29-105

**Published:** 2010-08-06

**Authors:** Ondrej Slaby, Jana Jancovicova, Radek Lakomy, Marek Svoboda, Alexandr Poprach, Pavel Fabian, Leos Kren, Jaroslav Michalek, Rostislav Vyzula

**Affiliations:** 1 Masaryk Memorial Cancer Institute, Department of Comprehensive Cancer Care, Zluty kopec 7, Brno, Czech Republic; 2 Masaryk Memorial Cancer Institute, Department of Oncological and Experimental Pathology, Zluty kopec 7, Brno, Czech Republic; 3 Babak Research Institute, University Cell Immunotherapy Center, Kamenice 5, Brno, Czech Republic; 4 Masaryk University, Faculty of Science, Department of Biochemistry, Kotlarska 2, Brno, Czech Republic; 5 University Hospital Brno, Department of Pathology, Faculty of Medicine, Masaryk University, Brno, Czech Republic

## Correction

After the publication of this research article [1], the authors noticed an error with Figure 1. Graph D which should have indicated miR-106b expression levels in renal parenchyma (RP) and renal cell carcinomas (RCC), was mistakenly displayed as a duplicate of Graph C. The corrected Figure 1 is provided here.

**Figure 1:**